# Circulating Serum Level of Visfatin in Patients with Endometrial Cancer

**DOI:** 10.1155/2018/8576179

**Published:** 2018-01-04

**Authors:** Aneta Cymbaluk-Płoska, Anita Chudecka-Głaz, Ewa Pius-Sadowska, Agnieszka Sompolska-Rzechuła, Bogusław Machaliński, Janusz Menkiszak

**Affiliations:** ^1^Department of Gynecological Surgery and Gynecological Oncology of Adults and Adolescents, Pomeranian Medical University in Szczecin, Al. Powstańców Wielkopolskich 72, 70-111 Szczecin, Poland; ^2^General Pathology Department, Pomeranian Medical University in Szczecin, Al. Powstańców Wielkopolskich 72, 70-111 Szczecin, Poland; ^3^Department of Mathematics Applications in Economy, West Pomeranian University of Technology, Al. Piastów 17, 70-310 Szczecin, Poland

## Abstract

Obesity is a well-known factor that leads to many diseases including endometrial cancer. The adipose tissue is a heterogeneous organ of internal secretion. Visfatin is a newly discovered protein produced by fat tissues. The purpose of this work was to evaluate serum level concentrations of visfatin in patients with endometrial cancer based on clinical progression and histopathological tumor differentiation. The diagnostic capabilities of visfatin protein in high differentiation (FIGO III and IV) from a lower (FIGO I and II) clinical stage and prognostic degree of cell differentiation (G1 versus G2, G2 versus G3) on the basis of the analysis of the area under the ROC curve are as follows: 0.87, 0.81, and 0.86. Significantly higher concentrations of visfatin have been observed in patients with invasion of the blood vessels (*p* = 0.02) and lymph node metastases (*p* = 0.01) in reference to the depth of infiltration of the endometrium (*p* = 0.004), as well as the size of the tumor (*p* = 0.003). Visfatin serum concentrations did not differ due to the invasion of the lymphatic vessels only. Visfatin seems to be a good marker of endometrial cancer progress. High visfatin serum level predicts poor prognosis in endometrial cancer patients.

## 1. Introduction

Lifestyle in developed countries has changed significantly in the last decades. The lack of physical activity and the easy access to high-calorie food contributed to the creation of an obese population [1]. Obesity is a well-known factor that conduces to many diseases including cancer [2, 3]. The adipose tissue is an inhomogeneous organ of internal secretion. Many changes are to be observed in it, including aromatization of androgen to estrogen, which affects, due to their hyperfunction, a high frequency of such estrogenic-related tumors as breast cancer and endometrial cancer. The adipose tissue discharges a variety of active proteins such as leptin, adiponectin, tumor necrosis factor, visfatin, resistin, omentin-1, and vaspin [4]. In 2009, Ye presented a study stating that patients characterized by long-lasting oxygen deficiency in the adipose tissue, as a consequence of the expansion of adipocytes, are affected by a long-standing inflammatory state of decreasing adiponectin, finally leading to the dysfunction of paracrine adipocytes [5]. In 2011, He et al. identified HIF-1 alpha and other mediators that cause hypoxia, conducing the release of free fatty acids from the adipose tissue. Free fatty acids directly affect the liver, and hence they are the causative factor of insulin immunity [6, 7]. A recent study in 2015 suggested that hypoxia induces an increased discharge of VEGF and increases visfatin gene (3TC-L1) expression of adipocytes; all this affects oncogenesis. In light of the above studies, we were given an impulse to conduct the research using visfatin [8, 9]. In our research, we would like to evaluate visfatin serum level in patients with different endometrial risk factors (obesity, diabetes mellitus type 2). However, the visfatin serum level in patients with endometrial cancer based on clinicopathological data is still unknown. In this study, we evaluated also serum concentrations of visfatin in patients with endometrial cancer based on clinical progression and histopathological tumor differentiation and whether serum visfatin levels can be associated with poorer patient prognosis.

## 2. Materials and Methods

### 2.1. Clinicopathological Data

The study included 128 patients who were admitted due to vaginal bleeding. All patients signed informed consent to participate in the study. The study protocol was approved by the Ethical Committee of Pomeranian Medical University.

Detailed division is presented in Tables 1 and 2.

Following surgical treatment and with the results of histopathological examination, patients were assorted into two groups:

1. Patients with endometrial cancer: *n* = 78.
2. Patients with normal endometrium: *n* = 63.

Among the group of patients with endometrial cancer, we identified the following:

1. 64 patients with endometrial cancer.
2. 14 patients with nonendometrial cancer.

Patients from the endometrial cancer group were divided according to tumor grading into subgroups G1 = 17, G2 = 41, and G3 = 20, as well as depending on clinical tumor staging:

(1a) FIGO I and II patients, *n* = 62.
(2a) FIGO III and IV patients, *n* = 16.

The study assessed the depth of myometrium infiltration, size of the tumor, invasion of blood vessels and lymphatic vessels, and metastasis to lymph nodes.  Table 3 provides the exact distribution of the individual subgroups.

### 2.2. Laboratory Measurements

Five milliliters of blood was collected from each patient (for the determination of visfatin levels on the occasion of routine preoperative testing) and centrifuged. The serum was subsequently frozen and stored at −70°C.

### 2.3. Multiplex Immunoassay

Visfatin concentrations were quantified in serum/plasma by multiplex fluorescent bead-based immunoassays (Luminex Corporation, Austin, TX, USA) using commercial Human Bio-Plex Pro Diabetes Assays (Bio-Rad, Hercules, CA, USA). 50 *μ*L of antibody capture bead solution was added to each well of the assay plate and the plate was washed two times with 100 *μ*L of a wash buffer. After washing, 50 *μ*L of each blank, standard, and sample were added to the plate, and the plate was incubated with agitation for 1 hour at room temperature and in the dark. After this step, the well was washed with 100 *μ*L of a wash buffer three times using a handheld magnet. 25 *μ*L of a detection antibody cocktail was pipetted to each well and the plate was sealed and incubated at room temperature for 30 minutes on a plate shaker. After washing, 50 *μ*L of a streptavidin-phycoerythrin mixture was added to the plate and incubated with agitation for 10 minutes in the dark. Finally, after washing, the microspheres in each well were resuspended in 125 *μ*L assay buffer and shaken at room temperature for 30 seconds. The plate was then read and analyzed on the Luminex analyzer and analyte concentrations were determined from five different standard curves showing MFI (median fluorescence intensity) versus protein concentration.

### 2.4. Statistical Analysis

Statistical analysis was performed using STATISTICA 10.0 PL program. The descriptive characteristic of the examined population of patients was prepared, determining minimum, maximum, mean, and median values. Because the distributions of the analyzed features to compare mean values are not normal distributions, median positional parameters were used as well as nonparametric tests (Kruskal-Wallis test and post hoc Dunn test for comparison of three groups and Mann–Whitney *U* test for comparison of two groups). For the selected groups, the receiver operating characteristic (ROC) curves were obtained and the area under the curve (AUC) was calculated with 95% confidence intervals according to the nonparametric method of DeLong. A *p* value of <0.05 was considered as statistically significant. The study variables were analyzed using the logistic regression model.

Logistic regression coefficients may be determined using the maximum likelihood method or the generalized least squares method. Due to the nonlinearity of the model in relation to the independent variables and parameters, the logistic model is transformed into a linear regression model using logarithmic transformation. To this end, the concept of odds ratio (OR) is introduced as the ratio between the likelihood of a particular event and the likelihood of that event not happening. Therefore, the odds ratio is used to express the factor of the increase or the decrease in the likelihood of a particular event upon a unit change in the independent variable (with fixed values of the remaining independent variables). Kaplan-Meier survival analysis and log-rank test were used to assess the impact of visfatin on overall survival and progression time. The following cut-off values were determined for baseline visfatin levels: median and level of 20.7 ng/ml. Single and multifactorial analysis was performed using the Cox regression model. Multifactorial Cox analysis parameters included age, FIGO, grading, median, and cut-off 20.7 ng/ml visfatin levels.

## 3. Results

### 3.1. Comparative Analysis of the Treatment Groups

The mean concentrations of individual proteins in serum are presented in Table 3. There is evidence of statistically significant lower mean concentrations of visfatin in patients with correct level of glycemia, without diabetes mellitus type 2 in relation to mean concentrations in patients with obesity or overweight, with an elevated level of glycemia, as well as diabetes mellitus type 2. Statistically significant correlations were found between WC patients and visfatin concentration, *r* = 0.882 for *p* = 0.002. The study has shown statistically significant differences in mean visfatin concentrations between patients with diabetes mellitus type 2 treated with metformin and patients with diabetes mellitus type 2 treated with insulin, respectively (21.3 ng/ml, 28.4 ng/ml). In contrast, the correlation between blood pressure and the mean values of visfatin protein was not found. The results are summarized in detail in Table 3.

We have found statistically significant differences in mean serum visfatin concentrations between a group of patients with endometrial cancer and patients with bleeding disorder and unchanged endometrium. The date was found among all analyzed women (*p* = 0.002) as well as after having taken into account the distribution depending on the hormonal status (premenopausal, *p* = 0.003; postmenopausal, *p* = 0.0002). The characteristics of the patients and the mean values of the test protein along with the status of hormonal distribution of patients are presented in Table 3.

### 3.2. Comparative Analysis due to the Risk Factors

**Figure 1:**
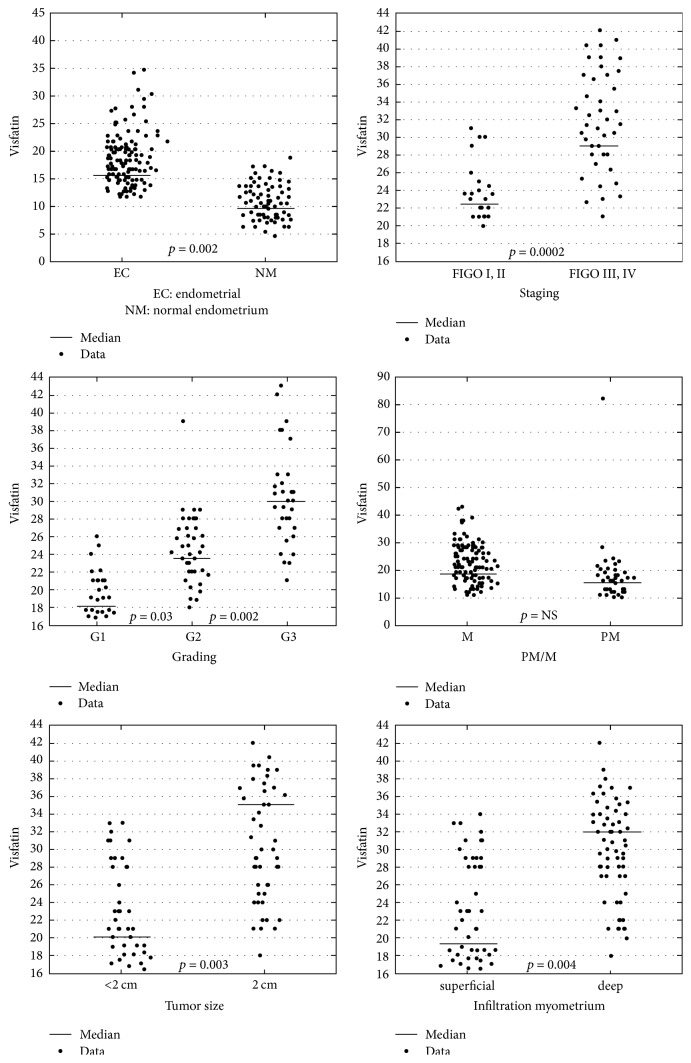
Visfatin serum levels comparison in individual groups.

Table 4 shows serum concentrations of visfatin due to prognostic factors together with comparisons. Having analyzed all currently recognized adverse prognostic risk factors of endometrial cancer, it has been stated that between two types of cancer (endometrioid endometrial cancer versus nonendometrioid endometrial cancer) there are no statistically significant differences. Visfatin serum concentrations did not differ due to the invasion of lymphatic vessels. However, statistically significant differences between the well-differentiated cancer group and a mean differentiated cancer group at mean concentration (17.3 ng/ml, 22.2 ng/ml) and between G2 and G3 (22.2 ng/ml, 31.8 ng/ml) have been proved (Figure 1). Significantly higher concentrations of visfatin have been observed in patients with highly advanced cancers versus lower advanced cancers, *p* = 0.0002, and in the case of the invasion of blood vessels, *p* = 0.02, and lymph node metastases, *p* = 0.01, in reference to the depth of infiltration of the endometrium, *p* = 0.004, as well as the size of the tumor, *p* = 0.003.

### 3.3. The ROC Analysis and Sensitivity and Specificity of the Test

**Figure 2:**
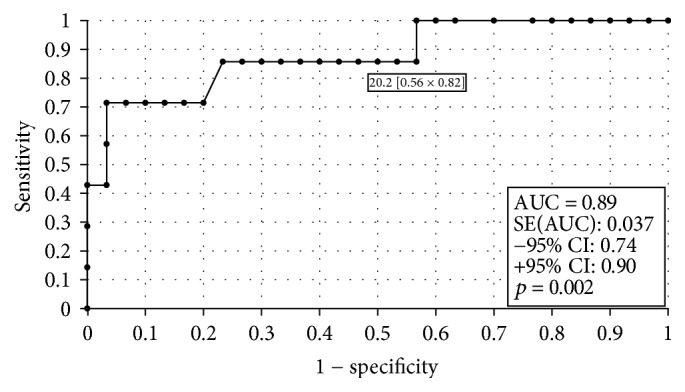
The ROC curves for visfatin proteins in women. The analysis compared endometrial cancer patients to patients with benign endometrial lesions.

**Figure 3:**
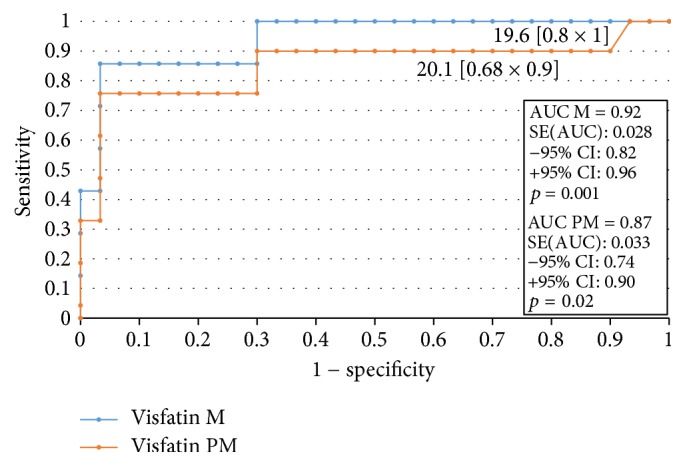
The ROC curves for visfatin proteins in women. The analysis compared endometrial cancer patients to patients with benign endometrial lesions depending on the hormonal status.

**Figure 4:**
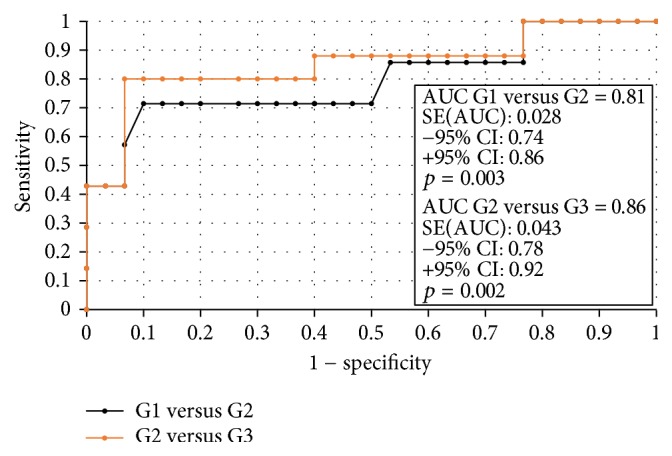
The ROC curves for visfatin proteins depending on endometrial cancer grading.

**Figure 5:**
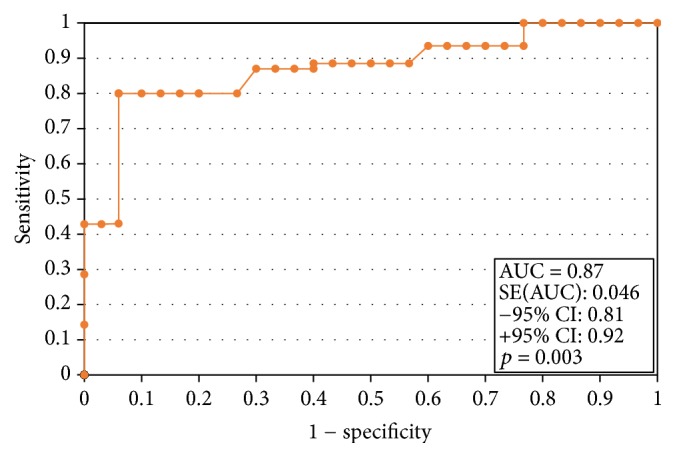
The ROC curves for visfatin proteins depending on endometrial cancer staging.

In order to evaluate the diagnostic value of visfatin, the ROC curves were established and the area under the ROC curve (AUC) was assessed. For visfatin, the AUC values were 0.89 for all the analyzed patients, 0.87 for premenopausal women, and 0.92 for postmenopausal women. The diagnostic capabilities of visfatin protein in high differentiation (FIGO III and IV) from a lower (FIGO I and II) clinical stage and prognostic degree of cell differentiation (V1 versus G2, G2 versus G3) on the basis of the analysis of the area under the ROC curve are as follows: 0.87, 0.81, and 0.86. This data has been presented in Figures 2–5. Having taken into account the hormonal status, the values of sensitivity and specificity for visfatin are presented in Table 6. We found greater sensitivity than specificity for visfatin in all analyzed women (88% versus 79%): for postmenopausal women, 90% versus 83%, in comparison with the sensitivity and specificity for premenopausal women, 85% versus 73%.

### 3.4. Multivariate Logistic Regression Analysis

Having applied multivariate logistic regression analysis for the risk of the development of endometrial cancer, in the final model, independent risk factors were found: glucose level, BMI, WC, and visfatin level. Classification determined on the basis of the cut-off values for visfatin (20.7 ng/ml) gives 94.38% correctly classified cases, out of which correctly classified “Yes” is 72.94% (sensitivity [95% CI] = 72.94% [48.81%; 91.02%]) and “No” is 97.74% (specificity [95% CI] = 97.74% [85.46%; 99.48%]) (Table 5).

### 3.5. Patients' Survival Time Evaluation Using the Kaplan-Meier Curves and Cox Proportional Hazards Regression

**Figure 6:**
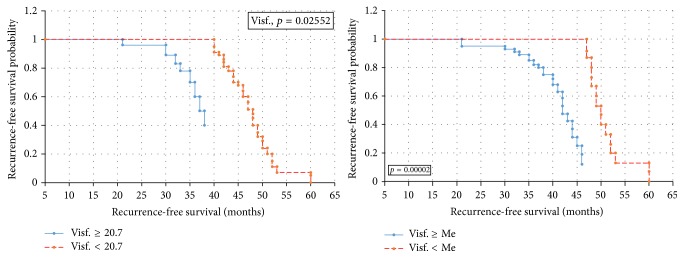
Kaplan-Meier recurrence-free survival curves for endometrial cancer patients based on serum visfatin level.

**Figure 7:**
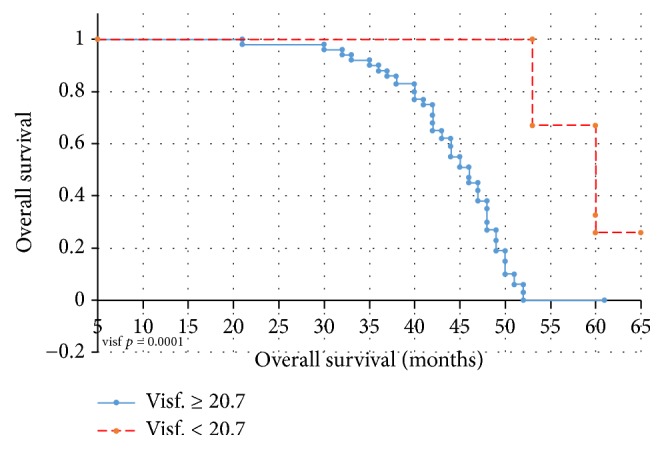
Kaplan-Meier overall survival curves for endometrial cancer patients based on serum visfatin level.

Statistical analysis performed using the Kaplan-Meier survival curves and log-rank test revealed that there are statistically significant correlations between the median and 20.7 ng/ml cut-off visfatin levels with recurrence-free survival of patients (*p* = 0.00002/*p* = 0.02552) (Figure 6). High baseline visfatin levels were shown to be correlated with shorter overall survival times in endometrial cancer patients (*p* = 0.0001) (Figure 7). Cox proportional hazards regression analysis with a single group variable revealed a correlation between the recurrence-free survival and clinical tumor staging (*p* = 0.0003). Univariate Cox regression analyses revealed correlations between overall survival and age (*p* = 0.038), tumor staging (*p* = 0.027), and above-median visfatin levels (*p* = 0.0056). In the RFS model, multivariate Cox regression analysis with age, grading, clinical stage, and above-median visfatin levels as the group variables revealed that patients with above-median visfatin levels had shorter RFS times compared to patients with below-median visfatin levels (*p* = 0.023, HR = 0.99). In the univariate OS model, both median visfatin level and cut-off baseline visfatin level (20.7 ng/ml) were significant variables. A correlation was demonstrated between median visfatin levels and overall survival of patients (*p* = 0.03, HR = 0.97). The higher the visfatin level above 20.7 ng/ml, the shorter the overall survival of patients.

## 4. Discussion

Obesity is a major health problem in the population in most highly developed countries. Approximately 40% of postmenopausal women have a problem with overweight or obesity. There is a known fact that certain cancers correlate with obesity; these include, for example, endometrial cancer [10, 11]. Diabetes mellitus type 2 and insulin resistance associated with it are well-known risk factors for endometrial cancer. New active biological factors that are secreted in the visceral adipose tissue and aid the formation of tumors are investigated. This factor may be visfatin. Our research has confirmed the positive correlation between visfatin and obesity/diabetes mellitus type 2 and evidence of neoplastic changes in every analyzed patient group. Visfatin as a cytokine was identified for the first time by Samal et al. in 1994 [12].

Sheu et al. emphasized that a significant weight reduction correlates with a decrease of serum levels of leptin and visfatin in patients by 17.2 and 50.2%, respectively, and with an increased level of adiponectin by 32.2% [13]. A meta-analysis conducted by Chang et al. proves that the increase of visfatin level is evident in obesity, diabetes mellitus type 2, and metabolic syndrome [14]. The characteristic common for the diseases mentioned above is the phenomenon of insulin resistance, whereby in a sequence of events, insulin resistance, hyperinsulinemia, the mechanism of downregulation, and the number of receptors for insulin decreased. It has also been shown that visfatin is an adipokine with proinflammatory and immunomodulatory properties.

It has the ability to activate human leukocytes, induces the synthesis of proinflammatory cytokines and adhesion molecules, regulates the maturation of leukocytes B, and inhibits apoptosis of neutrophils [15–17]. It also has a hepatoprotective effect in nonalcoholic fatty liver disease (NAFLD) [18]. It assumes a role in the regulation of obesity-related inflammation [19].

It is produced by a number of cells and its elevated concentration can be found in many acute and chronic inflammatory diseases [20]. The serum visfatin concentration is correlated with the metabolic syndrome [21].

Studies have shown that visfatin/PBEF is identical to nicotinamide (Nampt) phosphoribosyltransferase, which regulates the intracellular synthesis of adenine dinucleotide (NAD) from nicotinamide and thus energy processes in the cell [16]. In addition, visfatin increases the synthesis of nicotinamide mononucleotide (NMN), which stimulates the *β* pancreatic cells for insulin production.

As we know, visfatin stimulates the synthesis of TNF and IL-6, which could suggest its adverse effect on the development of insulin resistance [15].

In addition, by indirectly stimulating the synthesis of NF-*κ*b and increasing the production of free radicals, visfatin may additionally contribute to the rise of insulin resistance in obese patients [15, 22]. Nampt/visfatin acts both intracellularly and extracellularly, regulating many biological functions in the body, including the metabolism of NAD, carcinogenesis, inflammation, and stress.

Despite showing the multifunctionality of proteins, there are few reports emphasizing its role in carcinogenesis. Previous reports indicate that visfatin can function as a growth factor or as a cytokine in molecular mechanisms, including signaling pathways PI3K/Act, EQ1/2, and STAT3 [23, 24]. Exogenous administration of recombinant visfatin increases the proliferation of cells in human cells of breast cancer MCF-7 [22]. In a research, Lee et al. found that the high expression of visfatin in breast cancer tissue was associated with higher progression of breast cancer and poor prognosis [25]. Folgueira et al. also concluded that hyperexpression of visfatin may predict a poor response to doxorubicin-based chemotherapy for breast cancer [26].

The research by Wang et al. has revealed that there is an increased visfatin expression in endometrial cancer cell lines, promoting proliferation by activating the signaling pathways PI3K/Akt and MAPK/ERK1/2 [27]. Our research has proved a statistically significant increase of visfatin levels in patients with endometrial cancer compared to patients with normal endometrium. Similar results are presented by Ilhan et al. They showed the mean concentration of the EC group for visfatin at 14.9 ng/ml and in the control group at 8.1 ng/m [28]. In our study, the mean concentrations in both groups were slightly higher and were as follows: 15.9 ng/ml for the EC and 9.5 ng/ml for the control group. Evidence of the higher concentrations of visfatin protein in endometrial cancers is also presented in the studies of Avcioglu et al. [29–31]. Only Luhn et al., who evaluated the serum level concentrations of all adipokines in patients with endometrial cancer, proved the higher visfatin levels, but without statistical significance [32]. The authors themselves stressed that the results require confirmation due to the small number of patients tested. Tian et al. demonstrated that the level of visfatin expression in tissues corresponds to the serum concentration of visfatin in patients with endometrial cancer [31]. The role of visfatin in predicting clinical tumor stage was analyzed by Ilhan et al., by specifying it as an invasion of the myometrium [28]. Serum concentration of visfatin was far higher in patients with infiltration exceeding 1/2 of the myometrium. The cut-off point for visfatin for the clinical stage of IB tumor was 26.8 ng/ml. Ilhan et al. also investigated visfatin concentration depending on changes in the lymph nodes [28]. They found no statistically significant differences. In our study, the serum concentration of visfatin was higher but statistically insignificant. Serum concentration of visfatin in our study was evaluated also in occupied lymphatic and blood vessels as well as depending on the histopathological differentiation of the tumor. The higher mean serum concentration of visfatin has been found in the case of larger tumor size as lower histological differentiation of tumor and occupied lymphatic vessels.

Similar results are presented by Tian et al. who suggested that high levels of visfatin were associated with higher clinical staging and depth of myometrium infection. The same authors emphasized, however, that the expression of visfatin in the endometrium cancer tissue was not correlated with the degree of histopathological differentiation and lymph nodes metastasis. It may be a prognostic factor in patients with endometrial cancer. In our study, we found that high visfatin serum level will correlate with faster resumption and shorter survival time of patients with endometrial cancer. The same results were presented by Tian et al. who, analyzing the relationship between visfatin expression and total patient survival time, stated that high visfatin expression is believed to be associated with poor prognosis of patients, shorter survival time, and shorter time of recurrence.

Avcioglu et al. have demonstrated that the visfatin serum level can be treated as a risk factor for endometrial cancer on a par with patient age, BMI, and diabetes mellitus type 2 [29]. Using multivariate logistic regression analysis in our research for the development of endometrial cancer risk, in the final model, independent risk factors like glucose level, BMI, WC, and the visfatin level were found. The odds ratio of endometrial cancer when increasing the visfatin concentration of 1 ng/ml is 1.23.

## 5. Conclusion

1. Higher visfatin levels were found in obesity and type 2 diabetes. Both diseases are conducive to endometrial cancer.
2. Visfatin seems to be a good marker of endometrial cancer progression.
3. High serum visfatin levels can be associated with poorer patient prognosis.

## Figures and Tables

**Table 1:** Characteristics of patients with various risk factors of endometrial cancer.

Risk factor	*n*
PM/M	29/99

BMI < 25	24
BMI 25–30	38
BMI > 30	66

WC < 100	44
WC > 100	84

Glucose < 110	40
Glucose 110–126	36
Glucose > 126	52

DM-metformin	24
DM-insulin	28

Mean systolic HA < 140 mmHg	49
Mean systolic HA > 140 mmHg	79

PM: premenopausal; M: postmenopausal; BMI: body mass index; WC: waist circumference; DM-metformin: diabetes mellitus treated with metformin; DM-insulin: diabetes mellitus treated with insulin; HA: arterial hypertension.

**Table 2:** Concentration of visfatin in serum depending on the patient's histopathological diagnosis.

	Endometrial cancer	Normal endometrium
Age	54.6	52.9
Average height [cm]	166.9 ± 18.9	172 ± 17.2
Average weight [kg]	94.7 ± 16.5	89 ± 15.7
BMI	31.3 ± 5.2	29.8 ± 4.7
WC	106.5 ± 10.8	103.2 ± 8.99
HA	146/93	138/84
DM type 2	77.2%	55.2%

BMI: body mass index; WC: waist circumference; HA: arterial hypertension; DM type 2: diabetes mellitus type 2.

**Table 3:** Visfatin concentration in serum depending on risk factors for endometrial cancer.

	Mean	Median	Min.	Max.	SD	*p*
PM/M	14.9/17.1	15.3/18.4	7.1/8.4	20.9/23.3	1.08/1.28	NS
BMI < 25 BMI 25–30	9.2/14.3	10.1/16	6.8/5.9	18.2/31.2	2.01/3.3	0.02
BMI 25–30 BMI > 30	14.3/21.1	16/23.4	5.9/7.8	31.2/36.7	3.3/3.6	0.001
WC < 100/WC > 100	12.2/19.4	14.2/20.6	7.1/11.1	19.5/34.2	1.8/2.7	0.003
Glucose < 110/glucose 110–126	13.6/19.2	15.1/21.8	11.4/13.9	20.02/27.9	1.03/1.6	0.04
Glucose 110–126/glucose > 126	19.2/28.7	21.8/31.2	13.9/17.8	27.9/36.1	1.6/2.4	0.01
DM-metformin/DM-insulin	21.3/28.4	22.3/30.4	16.2/18.7	25.6/35.3	2.6/3.4	0.03
Mean systolic HA < 140 mmHg/mean systolic HA > 140 mmHg	18.2/23.4	19.7/24.5	15.1/16.2	22.2/28.4	1.9/2.3	NS

PM: premenopausal; M: postmenopausal; BMI: body mass index; WC: waist circumference; DM-metformin: diabetes mellitus treated with metformin; DM-insulin: diabetes mellitus treated with insulin; HA: arterial hypertension.

**Table 4:** Visfatin concentration in serum, depending on staging and grading group of endometrial cancer.

	Mean	Median	Min.	Max.	SD	*p*
Endometrioid/nonendometrioid	19.8/23.1	20.4/24.3	13.4/16.1	22.2/29.6	2.4/2.9	NS
G1/G2	17.3/22.2	18.3/23.9	13.2/10.2	19.9/31.3	1,8/3.7	0.03
G2/G3	22.2/31.8	23.9/33.8	10.2/14.6	31.3/41.2	3.7/4.2	0.002
FIGO I and II/FIGO III and IV	16.8/32.4	17.1/33.5	11.8/15.4	22.1/40.9	2.03/4.2	0.0002
Tumor size <2 cm or >2 cm	19.1/33.3	20.02/35.1	15.2/17.1	25.2/42.1	2.4/4.3	0.003
Infiltration of the myometrium: superficial/deep	18.4/30.6	19.1/31.8	11.2/17.3	25.1/38.7	2.2/3.6	0.004
Lymph vessels invasion +/−	25.5/22.3	27/23.1	17.2/16.5	29.1/26.6	2/2.2	NS
Vascular invasion +/−	29.8/21.4	30.3/22.3	16.7/19.1	39.2/30	4.1/2.8	0.02
Lymph nodes metastasis +/−	39.1/21.2	40.01/22.6	16.2/18.4	46.1/29.2	4.7/2.1	0.01

**Table 5:** Multivariate logistic regression analysis.

	OR	95% CI	*p*
BMI [kg/m^2^]	1.2	1.06–1.38	0.01
WC	1.03	0.98–1.18	0.02
Glucose level	0.79	0.66–0.97	0.32
DM type 2	0.87	0.73–1.03	0.56
Visfatin [ng/ml]	1.23	1.14–1.49	0.01
Age	1.06	0.97–1.18	0.04
HA	0.43	0.28–0.69	0.59

BMI: body mass index; WC: waist circumference; DM type 2: diabetes mellitus type 2; HA: arterial hypertension.

**Table 6:** Sensitivity and specificity for visfatin.

	Sensitivity	Specificity
Visfatin	88%	79%
Visfatin PM	85%	73%
Visfatin M	90%	83%

PM: premenopausal; M: postmenopausal.

## Abbreviations

OR: Odds ratio
95% CI: 95% confidence intervals
PM: Premenopausal
M: Postmenopausal
SD: Standard deviation
WC: Waist circumference
HA: Arterial hypertension
BMI: Body mass index
ROC: Receiver operating characteristic
AUC: Area under the curve
FIGO: Fédération Internationale de Gynécologie et d'Obstétrique (International Federation of Gynecology and Obstetrics)
G [1−3]: Grading
DM type 2: Diabetes mellitus type 2
DM-metformin: Diabetes mellitus treated with metformin
DM-insulin: Diabetes mellitus treated with insulin
OS: Overall survival
RFS: Recurrence-free survival.
